# Functional Connectivity of Auditory, Motor, and Reward Networks at Rest and During Music Listening

**DOI:** 10.3390/brainsci16010015

**Published:** 2025-12-22

**Authors:** Kai Yi (Kaye) Han, Jinyu Wang, Benjamin M. Kubit, Corinna Parrish, Psyche Loui

**Affiliations:** 1 Department of Psychology, Northeastern University, Boston, MA 02115, USA; han.kaiy@northeastern.edu; 2 Department of Music, Northeastern University, Boston, MA 02115, USA; wang.jinyu2@northeastern.edu (J.W.); b.kubit@northeastern.edu (B.M.K.); c.parrish@northeastern.edu (C.P.)

**Keywords:** music, functional connectivity, auditory, motor, reward, attention

## Abstract

Background/Objectives: Music engages multiple brain networks simultaneously, yet most studies examine these networks in isolation. Methods: We investigated functional connectivity among the auditory, motor, and reward networks during music listening in different contexts using fMRI data from two samples (*N* = 39 each): focused music listening and background music during cognitive tasks. ROI-to-ROI, seed-based, and graph theory analyses examined connectivity patterns among 46 regions spanning the three networks. Results: Both contexts showed enhanced within-auditory network connectivity compared to rest, suggesting that this is fundamental to music processing. However, between-network patterns diverged markedly. Background music listening during cognitive tasks preserved reward-motor coupling while reducing auditory-motor and auditory-reward connectivity. Focused music listening produced widespread negative correlations between motor regions and both the auditory and reward networks, potentially reflecting motor suppression in the scanner environment. Graph theory measures revealed context-specific hub reorganization: reward regions (nucleus accumbens, caudate) showed increased centrality during background music listening, while the amygdala and frontal orbital cortex were selectively enhanced during focused listening. Conclusions: These findings demonstrate that music engagement involves context-dependent network reorganization beyond simple attention effects. The same musical stimulus engages different neural mechanisms depending on concurrent cognitive demands, motor requirements, and listening goals. Enhanced within-auditory connectivity appears consistent across contexts, but between-network interactions are shaped by the broader cognitive-behavioral context. These results highlight the importance of considering ecological context when studying music processing and designing music-based interventions, as network connectivity patterns during music listening reflect complex interactions between task demands, attentional resources, and musical engagement rather than music processing alone.

## 1. Introduction

Engagement in music is intrinsically pleasurable and associated with positive health outcomes throughout the lifespan (see [1] for a review). Musical engagement can help regulate and express emotions (e.g., [2]), reduce stress-related symptoms (e.g., [3]), and promote social connection and prosociality (e.g., [4,5]), among many other benefits. As music is cost-effective, easily accessible, and very personalizable, there has been a rise in music-based interventions and music therapy for various health and neurological conditions, such as stroke, dementia, and Parkinson’s disease (see [6] for a review). Public and scientific interest in music’s therapeutic potential has burgeoned in recent years: with large-scale initiatives in the past decade such as the Sound Health Network, the NIH Music-Based Interventions Toolkit, the Music4Pain Research Network, and the Music Dementia Research Network, what was once a niche area of research has evolved into a multidisciplinary field spanning neuroscience, psychology, therapy, technology, and clinical medicine. Yet despite all these advances, understanding the mechanism of how music engages regions and networks throughout the brain is necessary to leverage the therapeutic potential of music for brain rehabilitation and overall well-being.

Music listening involves complex neurobiological mechanisms that engage multiple brain regions, including those involved in the auditory, reward, and motor networks. Musical stimuli, including monophonic and harmonized auditory sequences as well as instrumental and vocal music, activate various auditory regions, such as the superior temporal gyri (STG) and Heschl’s gyri (HG) [7,8]. As motor control plays a crucial role in music production (see [9,10] for a review), music involves a tight coupling between the auditory and motor networks. Listening to music and attuning to its rhythm and beat will spontaneously activate multiple regions of the motor network, including the premotor cortex, supplementary motor area (SMA), and basal ganglia [11,12]. Through the process of neuronal entrainment, it couples the motor and auditory networks with shared rhythmic oscillatory activity resembling the rhythm of the attuned musical stimulus [13,14,15].

Auditory-motor coupling is the strongest when listening to music that inspires movement [16,17]. Music that is groovy (i.e., that is perceived as having the pleasurable urge to move) elicits increased activity in the motor network, including the putamen and SMA, which are involved in movement and beat perception, as well as the prefrontal and parietal cortices, which are involved in movement initiation and preparation [18]. Similarly, listening to reggaeton, a genre known for its groovy dembow rhythmic patterns, elicited the highest auditory-motor activation compared to classical, electronic, and folk music [19]. Thus, motor system activity reflects the subjective feeling of grooviness and the simulation of covert body movement when listening to rhythms and beats [20].

Listening to enjoyable music generally stimulates and enhances the connectivity of the auditory-reward network, irrespective of a person’s musical expertise [21,22,23,24,25]. Changes in functional connectivity (FC) induced by TMS between the NAcc and both the frontal and auditory cortices predicted the degree of modulation in hedonic responses to music, suggesting that the NAcc plays a crucial role in music-induced reward and motivation [26]. Increased FC between the NAcc and the auditory cortices, amygdala, and ventromedial prefrontal regions predicted how rewarding a piece of music would be [27]. Individuals with music anhedonia (i.e., individuals who do not find music pleasurable) exhibited lower activity in the NAcc and reduced FC between the right auditory cortex and the ventral striatum, including the NAcc [28]. Additionally, white matter connectivity between the STG and areas associated with emotional and social processing in the insula and medial prefrontal cortex was related to individual differences in musical reward sensitivity [29]. Even in the absence of music, these two networks are intrinsically connected, and this between-network connectivity is preserved in healthy older adults and those with mild cognitive impairment ([30], but also see [31]).

While we recognize the roles of the auditory, reward, and motor networks during music listening, much remains unknown about how these three networks interact with each other. The few studies that have examined FC during music listening have predominantly focused on the connectivity between pairs of networks rather than all three simultaneously. By investigating the interactions among all three networks, we can gain a better understanding of the overall organization of the brain while processing music, particularly how the motor regions contribute to both the pleasurable nature of music and the auditory-reward circuitry. Furthermore, by moving beyond simple activations in specific regions to more summative or network-based metrics, graph theory can capture patterns of connectivity and network organization across brain regions, revealing integrative, distributed, and emergent properties of neural systems.

Moreover, music is experienced in various contexts—sometimes as the primary focus, sometimes accompanying other activities. Understanding network connectivity in these different contexts is essential for understanding the neural basis of music listening. Prior work in EEG has observed differences in the onset and amplitude of Event-Related Potential (ERP) components between foreground and background music listening [32], suggesting that attention influenced the underlying neural processing of complex musical stimuli. One study [33] found differences in event-related synchronization between passive and active listening conditions but only for a well-known pop song and not for an unfamiliar classical music piece. Using behavioral and neuroimaging methods, another study [34] investigated how background music with fast and slow amplitude modulations affected sustained attention, measured by performance on the Sustained Attention Response Task (SART). Participants who listened to music with fast amplitude modulations first outperformed those who listened to music with slow amplitude modulations and pink noise. Results suggest a complex relationship between music, attention, and the brain.

By characterizing functional connectivity within and between brain networks under conditions of foreground and background music listening, the current work seeks to uncover the interactions across the auditory, motor, and reward networks across a few different task settings under which music is commonly experienced. In one setting, participants listened to musical excerpts (in the foreground) and evaluated them for liking and familiarity. In another setting, participants completed attention and working memory tasks while music was playing (in the background). In both settings, participants also completed resting-state functional MRI scans in which there was no task; these resting-state scans served as a no-music no-task control across all participants.

In the present study, we conduct a preregistered secondary analysis of fMRI data for FC under different music listening contexts relative to resting-state data obtained from the same participants. We aim to identify FC patterns of the auditory, reward, and motor networks underlying music listening. As limited fMRI studies have explicitly examined variations in functional connectivity during attentive foreground versus inattentive background music listening, another goal of the current work is to identify potential FC differences in the three networks of interest that could be associated with foreground versus background listening. We hypothesize that (i) there will be increased FC within and between reward-associated brain regions and the auditory and motor networks during music listening compared to rest. We also hypothesize that (ii) FC patterns between the three networks will differ across music listening contexts with varying levels of attentional engagement. This secondary analysis examines existing datasets from two independent studies involving music listening. Although attentional engagement was not experimentally manipulated, these datasets reflect ecologically valid music listening contexts with natural variations in attention.

## 2. Materials and Methods

### 2.1. Participants

We analyzed two samples of Northeastern University students from two separate studies. The first sample (foreground music group) consisted of *N* = 39 young adults aged 18 to 25 (*M* = 20.18 years, *SD* = 3.09 years; 29 identified as female, 9 as male, and 1 as non-binary). We excluded one participant due to extremely high mean motion values. The second sample (background music group) consisted of *N* = 39 young adults aged 18 to 25 (*M* = 19.21, *SD* = 1.88; 29 identified as female, 7 as male, and 3 as non-binary). We excluded one participant due to extremely high maximum global signal change values. All participants met the following inclusion criteria: (1) were 18 years of age or older, (2) had normal hearing, and (3) passed an MRI safety screening. They were compensated with either payment or course credit. Both studies were approved by the Northeastern University institutional review board and preregistered at https://osf.io/d6j7e/overview (accessed on 10 September 2025).

### 2.2. Procedures

#### 2.2.1. Study 1: Foreground Music Group

Participants took part in a music listening fMRI task, where they listened to 20 s musical clips. These clips included participant-selected music (6 out of 24 stimuli), well-known Western songs from various genres chosen by the researchers (10 out of 24 stimuli), and music composed using the novel Bohlen-Pierce scale (8 out of 24 stimuli). A list of all chosen song stimuli is provided in Supplementary Materials S1. The order of the clips was randomized. After listening, participants had two seconds to rate each clip based on how much they liked and were familiar with it using a 4-point scale, with 1 being Hate/Very unfamiliar and 4 being Love/Very familiar (for more details, see [21]).

They also completed a face-name association memory test [35] without any music. They learned the faces and names of 30 individuals (16 females and 14 males) and were then tested on 14 of these individuals (7 females and 7 males). Each face-name pair was shown once for 4.75 s, followed by a 1.9 s central fixation crosshair, except for one female and one male, whose pairs were presented four times across the four trial blocks. During the testing phase, participants saw one of the 30 previously presented individuals’ faces alongside two names: the correct associated name and a distractor name linked to another face from the learning phase. The placement of the correct and distractor names was randomized across trials.

#### 2.2.2. Study 2: Background Music Group

Participants completed the 2-Back Working Memory (WM) and Sustained Attention to Response tasks (SART) while listening to either theta-frequency amplitude-modulated or unmodulated background music. From a larger library of 30 well-known Western songs from the Billboard Top 100 s, each participant was randomly assigned a list of 12 songs. For each task, they listened to 6 songs (three of them modulated and the remaining three unmodulated). Thus, there were a total of 6 modulated songs and 6 unmodulated songs over the course of both tasks (see Supplementary Materials S1 for a list of songs). Each song clip lasted 60 s, with task stimuli presented at the beat rate of each song. Each trial lasted between 975 ms and 1250 ms depending on the song’s inter-onset interval. Task order (i.e., WM or SART first) and type of music (i.e., modulated or unmodulated music first) were counterbalanced. For all 30 songs, participants also provided liking and familiarity ratings using a 5-point scale, with 0 being Unliked/Very unfamiliar and 4 being Very Liked/Very familiar.

In the WM task, participants were presented with a sequence of 15 letters (from A to T) one at a time, and their task was to indicate with a button press whether the current letter was the same as the letter presented two trials earlier. For SART, participants saw a sequence of numbers (0 through 9), and their task was to click the button for every number except for a specified target number (3).

### 2.3. fMRI Data Acquisition and Analysis

All images were acquired using a Siemens Magnetom 3T MR scanner (Siemens Healthineer, Malvern, PA, USA) with a 64-channel head coil at the Northeastern University Biomedical Imaging Center. For the foreground music group, task fMRI data used continuous acquisition with a repetition time (TR) of 475 ms. Specifically, 672 and 196 volumes were collected for the learning and testing phase of the face-name task, for a total acquisition time of 5 min 26 s and 1 min 40 s, respectively. For the music listening task, 1440 volumes were collected, for a total acquisition time of 11 min 31 s. Forty-eight interleaved transverse slices (slice thickness = 3 mm, phase encoding [PE] direction = anterior to posterior) were acquired as multiband gradient echoplanar images (EPI) functional volumes covering the whole brain (field of view = 240 mm, resolution = 3 mm isotropic, TR = 475 ms, time echo [TE] = 30 ms, flip angle = 40° [face-name tasks]/60° [music listening task]). The resting-state scan followed the same parameters (flip angle = 60°) and included 947 continuous scans, for a total scan length of 7 min 37 s. Structural images were acquired using a high-resolution magnetization prepared rapid gradient echo (MPRAGE) sequence, with one T1 image acquired every 2500 ms, for a total task time of 8 min 22 s. Sagittal slices (0.8 mm thick, PE direction = anterior to posterior) were acquired covering the whole brain (field of view = 256 mm, resolution = 0.8 mm isotropic, TR = 2500 ms, TE 1 = 1.81 ms, TE 2 = 3.6 ms, TE 3 = 5.39 ms, TE 4 = 7.18 ms, flip angle = 8°, IPAT mode = GRAPPA 2×), as described in [21]. Spin-echo volume pairs were also acquired matching the BOLD EPI slice prescription and resolution in opposing PE directions (anterior to posterior and posterior to anterior) for susceptibility distortion correction (TR = 8290 ms, TE = 69.0 ms, acquisition time = 33 s each).

For the background music group, task fMRI data used continuous acquisition for 768 volumes per task (1536 volumes in total), with the same TR (475 ms), for an acquisition time of 6 min 12 s per task (12 min 24 s in total). Forty-eight interleaved transverse slices (slice thickness = 3 mm, PE direction = anterior to posterior, multiband acceleration factor = 8) were acquired as EPI functional volumes covering the whole brain (field of view = 240 mm, resolution = 3 mm isotropic, TR = 475 ms, TE = 30 ms, flip angle = 41°). The resting-state scan followed the same parameters and included 947 continuous scans, for a total scan length of 7 min 37 s. Structural images were also acquired using a high-resolution MPRAGE sequence, with one T1 image acquired every 2500 ms, for a total task time of 8 min 22 s. Sagittal slices (0.8 mm thick, PE direction = anterior to posterior) were acquired covering the whole brain (field of view = 256 mm, resolution = 0.8 mm isotropic, 208 slices, TR = 2500 ms, TE 1 = 1.81 ms, TE 2 = 3.6 ms, TE 3 = 5.39 ms, TE 4 = 7.18 ms, flip angle = 8°, IPAT mode = GRAPPA 2×). Lastly, spin-echo volume pairs were acquired matching the BOLD EPI slice prescription and resolution in opposing PE directions (anterior to posterior and posterior to anterior) for susceptibility distortion correction (TR = 8290 ms, TE = 69.0 ms, acquisition time = 33 s each).

All fMRI data were preprocessed using FMRIPrep (Version 23.1.4) [36] and the CONN Toolbox [37]. Functional and anatomical data were preprocessed using a modular preprocessing pipeline [38], including functional realignment and unwarp with correction of susceptibility distortion interactions, functional centering, functional slice time correction, functional outlier detection using the ARtifact detection Tools (ART) software package, functional direct segmentation and normalization to the Montreal Neurological Institute (MNI) template, structural centering, and structural segmentation and normalization to MNI template, and functional smoothing using spatial convolution with a Gaussian kernel of 8 mm full width half maximum [39]. Functional data was further denoised using a standard pipeline [38], including the regression of potential confounding effects characterized by white matter and CSF timeseries (10 CompCor noise components each), motion parameters and their derivatives (12 factors), outlier scans and their derivatives (below 14 factors), session and task effects and their derivatives (2 factors each), and linear trends within each functional run (2 factors), followed by high-pass frequency filtering of the BOLD timeseries above 0.008 Hz.

### 2.4. ROI-to-ROI Analyses

Using the CONN Toolbox, ROI-to-ROI connectivity matrices were estimated characterizing the patterns of functional connectivity with 46 ROIs of the auditory, reward, and motor networks. Specifically, we included 18 auditory ROIs (all subdivisions of bilateral superior temporal gyri [STG], middle temporal gyrus [MTG], inferior temporal gyri [ITG], and Heschl’s gyri [HG]) and 18 reward ROIs (anterior cingulate [ACC], posterior cingulate [PCC], bilateral insular cortex [IC], frontal orbital cortex [FOrb], caudate, putamen, pallidum, hippocampus, amygdala, and NAcc), as defined by [30]. We chose 10 ROIs for the motor network, including bilateral precentral (preCG) and postcentral gyri (postCG), SMA, and superior and middle frontal gyri (SFG and MFG). For further exploratory analyses, we also included 32 asymmetrical cerebellar region parcellation ROIs from [40].

We contrasted each task with rest and between each other (Foreground music group: Music Listening task > Rest, Music Listening task > Face-name task, Face-name task > Rest; Background music group: SART > Rest, WM > Rest, SART > WM). We included the face-name task in our contrasts as a control to determine whether any observed motor activity was attributable to general task-related processes (e.g., button presses and cognitive demand). As exploratory analyses, we compared the two background music tasks within and between conditions to examine potential effects of music modulation on FC; these comparisons revealed no significant modulation-related differences. All these main effect contrast analyses included participant age, gender, musical reward sensitivity measured by the Barcelona Musical Reward Questionnaire (BMRQ), and average liking and familiarity ratings as covariates of no interest. The connection threshold was *p* < 0.05 (False Discovery Rate [FDR] corrected), and the cluster threshold was *p* < 0.05 (FDR corrected based on Network-Based Statistics [NBS]).

### 2.5. Seed-Based Connectivity Analyses

We also conducted seed-based connectivity analyses using CONN, using the above defined task versus rest contrasts and auditory, reward, and motor network ROIs as seeds. Specifically, we examined the STG and HG for the auditory network, the NAcc and IC for the reward network, and all 10 ROIs for the motor network. Just like the ROI-to-ROI analyses described above, we ruled out confounding effects of within-group age, gender, and musical reward sensitivity by including them as covariates of no interest. All voxel and cluster thresholds were set at *p* < 0.05 FDR corrected.

### 2.6. Graph Theory Analyses

Additionally, to assess how music listening modulates the topological organization of functional brain networks, we looked at within- and between-task graph theory measures for all our chosen ROIs, including degree (i.e., the number of connections from a node), clustering coefficient (i.e., the cliquishness of a node, or the number of shared neighboring nodes each node has), betweenness centrality (i.e., the number of shortest paths that contains a given node), and local (i.e., the average inverse shortest path length of node neighborhoods) and global efficiency (i.e., the average inverse shortest path length in the network) [41]. We selected adjacency matrix thresholds of cost = 0.15 for between-task measures and correlation coefficient = 0.45 for within-task measures.

## 3. Results

### 3.1. Behavioral Ratings

#### 3.1.1. Study 1: Foreground Listening Group

On a 4-point scale, participants rated their own selected music highest in both liking (*M* = 3.97) and familiarity (*M* = 3.86). Experimenter-selected well-known pieces received moderate ratings (liking: *M* = 2.77; familiarity: *M* = 2.34), whereas experimenter-selected novel pieces were liked the least (*M* = 1.90) but showed moderate familiarity (*M* = 2.72). The relationship between liking and familiarity ratings and engagement of auditory–reward networks has been examined in several previous publications from our lab [21,42,43].

#### 3.1.2. Study 2: Background Listening Group

On a 5-point scale, the average liking and familiarity ratings across all 30 songs were 2.40 and 3.14, respectively. When examined by task, ratings were similar for SART (liking: *M* = 2.38; familiarity: *M* = 3.10) and WM (liking: *M* = 2.43; familiarity: *M* = 3.19). When examined by modulation condition, ratings were again similar for modulated (liking: *M* = 2.43; familiarity: *M* = 3.14) songs and unmodulated songs (liking: *M* = 2.38; familiarity: *M* = 3.15).

### 3.2. ROI-to-ROI Analyses

We first looked at within-task FC for both resting states and all tasks (i.e., music listening task, SART and WM combined, and face-name task) to ensure that our chosen ROIs for each network are intrinsically connected. As expected, most ROIs were positively connected to other within-network ROIs (see Supplementary Materials S2 for all within-task ROI-to-ROI connectivity matrices).

#### 3.2.1. Study 1: Foreground Listening Group

**Figure 1:**
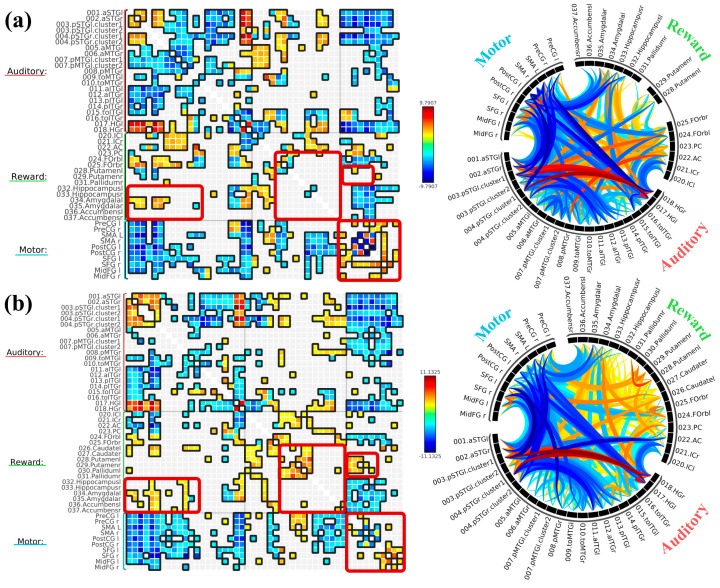
ROI−to−ROI connectivity graphs with the (**a**) Music Listening Task > Rest contrast and (**b**) Music Listening Task > Face−name Task contrast as matrices (**left**) and as rings (**right**). For both graph types, warmer colors (red−yellow) indicate higher FC between two given ROIs during the music listening task, and cooler colors (green−blue) indicate higher FC between two given ROIs during rest or the face-name task. The connection threshold was *p* < 0.05 FDR corrected, and cluster threshold was *p* < 0.05 (FDR corrected−Network Based Statistics [NBS]). Red boxes highlight major differences between the two contrasts, particularly reward ROIs (hippocampus, pallidum, caudate, NAcc).

For the music listening task and rest contrast, we found statistically positive bivariate correlations within most regions of the auditory network, including the bilateral anterior and posterior STG and HG. However, bilateral MTG and ITG were mostly negatively correlated to other auditory ROIs. Bilateral IC was negatively correlated with HG but positively correlated with bilateral anterior and left posterior MTG. ACC was negatively correlated with bilateral HG and posterior STG but positively correlated with bilateral MTG (especially left temporooccipital MTG) and temporooccipital ITG. PCC was negatively correlated with left anterior STG but positively correlated with temporooccipital MTG. Both bilateral FOrb and amygdala were positively correlated with STG, posterior and anterior MTG, and bilateral HG while negatively correlated with temporooccipital MTG and ITG. Bilateral putamen was negatively correlated with bilateral posterior STG. Bilateral hippocampus was positively correlated with bilateral HG and negatively correlated with temporooccipital ITG. Most motor ROIs were negatively correlated with auditory and reward ROIs, except for: bilateral SMA with PCC and ACC, bilateral precentral gyri with temporooccipital ITG, right precentral gyrus with temporooccipital ITG, right postcentral gyrus with PCC, bilateral SFG with left IC, bilateral midFG with left anterior ITG, and left midFG with ACC. All ROI-to-ROI results are displayed in Figure 1a.

Then, we contrasted the music listening task with the face-name task. There is a similar trend of enhanced FC within the auditory network and reduced FC outside of the auditory network (particularly with the motor network), as well as reduced reward-motor FC during the music listening task compared to the face-name task. One prominent difference was enhanced FC within the reward network, as well as between some auditory-reward ROI pairs. Specifically, the bilateral NAcc was positively correlated with posterior STG and MTG, and the bilateral caudate was positively correlated with left anterior STG and right posterior STG. Another difference was enhanced reward-motor FC, specifically bilateral pallidum and caudate with SMA and precentral gyri. All ROI-to-ROI results are displayed in Figure 1b.

#### 3.2.2. Study 2: Background Listening Group

**Figure 2:**
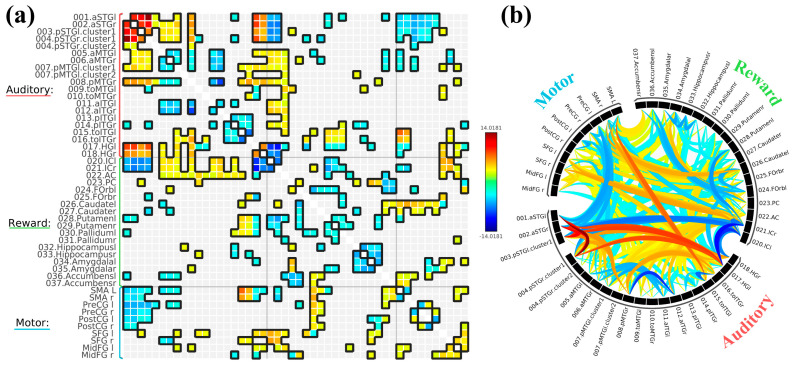
ROI−to−ROI connectivity graphs with the contrast SART and WM combined > Rest in matrix−form (**a**) and ring−form (**b**). For both graphs, warmer colors (red−yellow) indicate higher FC between two given ROIs during task, and cooler colors (green−blue) indicate higher FC between two given ROIs during rest. The connection threshold was *p* < 0.05 FDR corrected, and the cluster threshold was *p* < 0.05 (FDR corrected−Network Based Statistics [NBS]).

We first contrasted SART with WM but found no significant differences, so we decided to statistically treat them as one task (i.e., by taking the average of both SART and WM) to contrast with rest. There were similar patterns again, with positive correlations within most regions of the auditory network, including the bilateral STG, MTG, and HG, except for the ITG ROIs. Bilateral STG and HC were again negatively correlated to bilateral IC. MTG was negatively correlated to the left NAcc and caudate but positively correlated to the PCC, ACC, and bilateral IC. ITG ROIs were negatively correlated to the hippocampus and caudate but positively correlated to the ACC and PCC. The temporooccipital ITG were also positively correlated to the pallidum, putamen, caudate, and FOrb. Bilateral STG and HG were negatively correlated with bilateral SMA. The bilateral precentral and postcentral gyri were also negatively correlated with STG and MTG. The left SFG and midFG were positively correlated with bilateral HG, posterior MTG, and temporooccipital ITG. The right SFG was negatively correlated with anterior MTG and positively correlated with temporooccipital ITG, and the right midFG was negatively correlated with left HG and right posterior ITG. The reward ROIs, especially the NAcc and caudate, were also mostly positively correlated to the motor ROIs. All ROI-to-ROI results are displayed in Figure 2.

In all, these findings suggest that when individuals listen to background music while concurrently completing cognitive tasks, the auditory network had higher within-network connectivity and reduced out-of-network connectivity (auditory-reward and auditory-motor) compared to rest.

### 3.3. Seed-Based Connectivity Analyses

#### 3.3.1. Study 1: Foreground Listening Group

**Figure 3:**
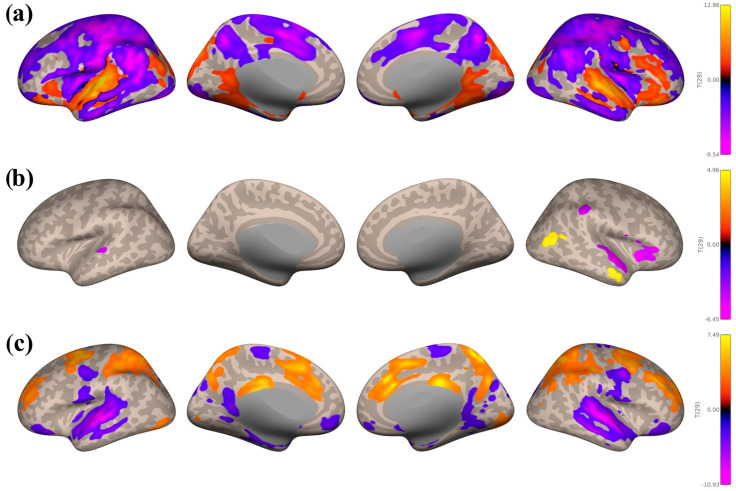
Seed−based connectivity maps with the Music Listening Task > Rest contrast, taking the average of seeds within the auditory network (**a**), the reward network (**b**), and the motor network (**c**). Seed-based connectivity during music listening showed consistent patterns across both contrasts. The auditory network had widespread negative connectivity in motor and occipital regions and positive connectivity localized to auditory cortex. The reward network was specifically positively correlated to the right aMTG but negatively correlated to other auditory regions. The motor network exhibited negative connectivity in occipital and precuneus areas but positive connectivity in frontal and motor regions (SFG, precentral gyri, ACC, midFG, SMA). All voxel and cluster significance thresholds were set to *p* < 0.05 FDR corrected.

**Figure 4:**
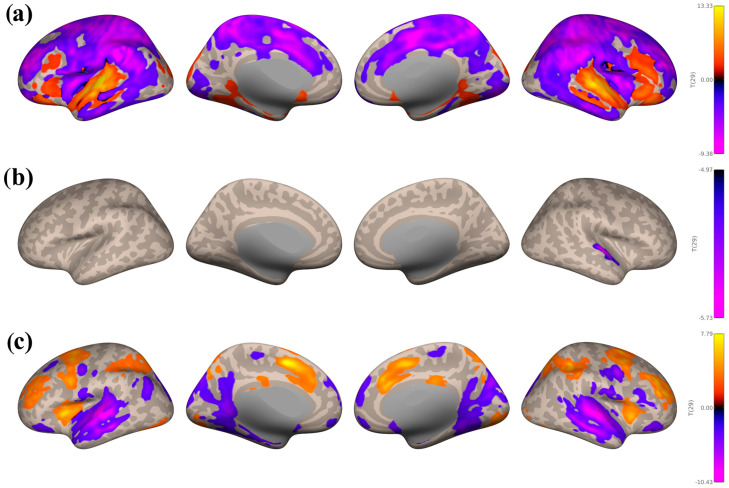
Seed−based connectivity maps with the Music Listening Task > Face-name Task contrast, taking the average of seeds within the auditory network (**a**), the reward network (**b**), and the motor network (**c**). There were similar trends of widespread out-of-network connectivity from the auditory network. All voxel and cluster significance thresholds were set to *p* < 0.05 FDR corrected.

There were similar seed-based connectivity trends in the Music Listening Task > Rest (Figure 3) and Music Listening Task > Face-name Task (Figure 4) contrasts. Seed-based connectivity patterns from the auditory network are shown in Figure 3a and Figure 4a below. Large negative clusters were found within the motor network, including the precentral and postcentral gyri, SFG, and midFG, followed by the frontal pole, lateral occipital cortex, precuneous cortex, and ACC. Smaller negative clusters were found within the superior lobule, angular gyri, and PCC. Large positive clusters were seen in the planum temporale, FOrb, STG, and HG. Smaller positive clusters were seen in the occipital pole.

There were smaller and fewer clusters for the reward network overall, with negative clusters predominantly in the planum temporale, planum polare, and HG (see Figure 3b and Figure 4b). The Music Listening Task > Rest contrast also had small negative clusters within anterior supramarginal gyri, as well as small positive clusters within the right anterior MTG.

As for the motor network (see Figure 3c and Figure 4c), there were large negative clusters in the lateral occipital cortex, precuneous cortex, lingual gyri, and occipital pole. Smaller negative clusters were found in auditory areas (left posterior MTG and STG, anterior MTG, and HG), as well as the planum temporale, temporal pole, FOrb, and planum polare. Large positive clusters were seen in the SFG, precentral gyri, ACC, paracingulate gyri, midFG, and SMA.

#### 3.3.2. Study 2: Background Listening Group

**Figure 5:**
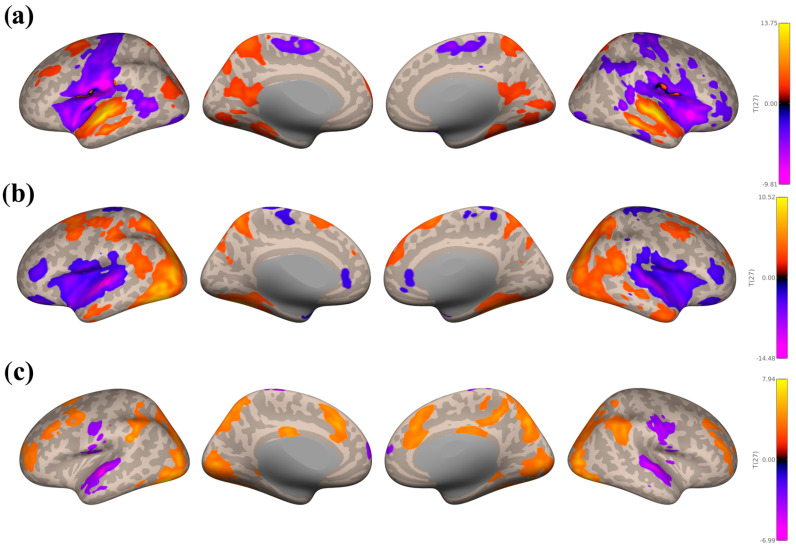
Seed−based connectivity maps with the contrast SART and WM combined > Rest, taking the average of seeds within the auditory network (**a**), the reward network (**b**), and the motor network (**c**). All voxel and cluster significance thresholds were set to *p* < 0.05 FDR corrected.

Seed-based connectivity patterns from the auditory network are shown in Figure 5a below for the SART and WM combined > Rest contrast. Large negative clusters were found within the reward (bilateral IC and putamen) and motor (bilateral precentral and postcentral gyri and SMA) regions. Large positive clusters were found in multiple auditory regions (MTG and STG, temporal pole, and HG), the precuneous cortex, and planum polare. There were smaller positive clusters in the lateral occipital cortex and cingulate gyri.

As for the reward network (see Figure 5b), there were large positive clusters covering the superior and inferior lateral occipital cortex and SFG, and slightly smaller clusters in the ITG and MTG. Negative clusters were found in the IC, central opercular cortex, frontal and temporal pole, planum temporale, and putamen.

Lastly, for the motor network (see Figure 5c), there were large positive clusters in the lateral occipital cortex, precuneous cortex, supramarginal gyrus, and occipital fusiform gyrus. Negative clusters were found in precentral and postcentral gyri, planum temporale, and STG.

### 3.4. Graph Theory Analyses

Lastly, we computed graph theory measures of degree, clustering coefficient, betweenness centrality, local efficiency, and global efficiency. We only reported between-task contrasts due to length constraints.

#### 3.4.1. Study 1: Foreground Listening Group

**Figure 6:**
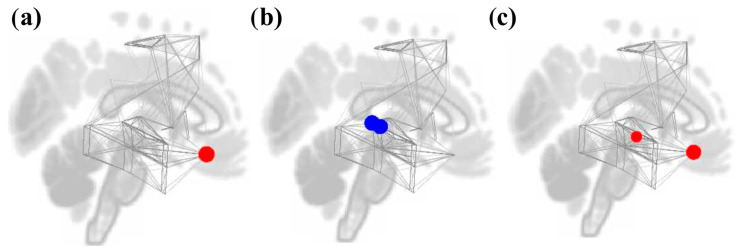
Node graphs for the Music Listening Task > Rest contrast depicting graph theory measures using an adjacency matrix threshold of cost = 0.15. Red dots represent positive statistics, while blue dotes represent negative statistics. Node degree increased in the right FOrb (**a**). Betweenness centrality (**b**) decreased in the bilateral posterior STG. Global efficiency (**c**) increased in both right FOrb and posterior STG.

For the Music Listening Task > Rest contrast, we only see a significant increase in node degree for the right FOrb. The bilateral posterior STG had significant decreases in betweenness centrality. There were no significant results for clustering coefficient and local efficiency. Lastly, global efficiency was significantly increased in the right FOrb and right posterior STG. Results are shown in Table 1 and Figure 6.

**Figure 7:**
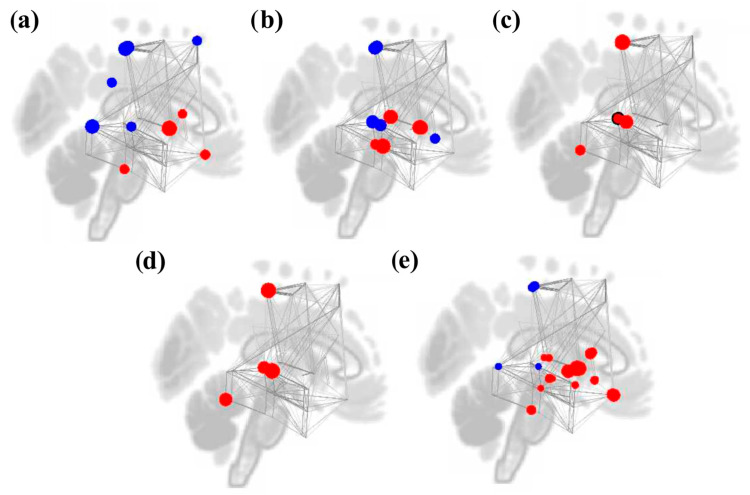
Node graphs for the Music Listening Task > Face-name Task contrast depicting graph theory measures using an adjacency matrix threshold of cost = 0.15. Red dots represent positive statistics, while blue dotes represent negative statistics. Increases in degree (**a**) were seen in reward ROIs, while decreases were seen in motor and other reward ROIs. Betweenness centrality (**b**) decreased in bilateral postcentral gyri and increased in most reward and auditory ROIs, except for bilateral posterior STG and left NAcc. Clustering coefficient (**c**) and local efficiency (**d**) increased in all auditory and motor ROIs. Global efficiency (**e**) increased in most reward and auditory ROIs but decreased in bilateral postcentral gyri.

For the Music Listening Task > Face-name Task contrast, significant increases in node degree were seen in the reward ROIs, specifically the right putamen, left FOrb, and left caudate but not PCC. Degree decreases were seen in the auditory (right temporooccipital MTG and right posterior STG) and (bilateral postcentral gyri and right SFG) motor ROIs. Betweenness centrality decreased in the bilateral postcentral gyri and increased in reward ROIs (bilateral putamen and IC). Betweenness centrality also decreased in the left posterior STG but increased in the bilateral posterior MTG and right HG. Clustering coefficient and local efficiency increased in the left temporooccipital ITG, bilateral postcentral gyri, and bilateral posterior STG. Global efficiency increased in the reward and auditory ROIs but decreased in the bilateral postcentral gyri, right temporooccipital MTG, and right posterior STG. Results are shown in Table 2 and Figure 7.

#### 3.4.2. Study 2: Background Listening Group

Compared to rest, cognitive task performance (SART and WM) with music was associated with significant decreases in node degree in the reward (bilateral IC) and auditory (right posterior and anterior ITG) networks, as well as the left SMA. However, several reward ROIs also showed significant increases in degree, including the bilateral NAcc, left caudate, right pallidum, and left midFG. There were no significant results for betweenness centrality.

**Figure 8:**
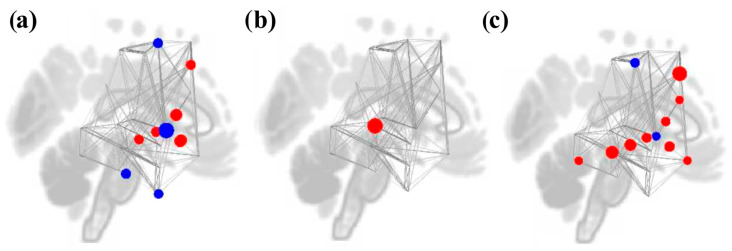
Node graphs for the SART and WM combined > Rest contrast depicting graph theory measures using an adjacency matrix threshold of cost = 0.15. Red dots represent positive statistics, while blue dotes represent negative statistics. Decreases in degree (**a**) were seen in bilateral IC and auditory ROIs, while increases were seen in mostly reward ROIs. Clustering coefficient and local efficiency (**b**) increased in only the right pSTG. Global efficiency (**c**) increased in multiple auditory, reward, and motor ROIs but decreased in left preCG and right IC.

Clustering coefficient and local efficiency were significantly increased in the right pSTG during music listening relative to rest. Significant increases were also observed for global efficiency. Specifically, the bilateral midFG for the motor network; the left pMTG, right pSTG, and right temporooccipital ITG for the auditory network; and the right pallidum, bilateral NAcc, left FOrb, left caudate, and ACC for the reward network. Only the left preCG and right IC saw decreases in global efficiency. Results are shown in Table 3 and Figure 8.

## 4. Discussion

We characterized FC patterns among auditory, motor, and reward networks during music engagement in two ecological contexts: music listening during cognitive task performance and focused music listening. While both contexts revealed enhanced within-auditory network connectivity during music processing, they showed distinct patterns of between-network interactions that likely reflect the combined influence of task demands, cognitive load, motor requirements, and music processing itself. These findings provide novel insights into the tripartite interaction of these networks during music engagement and demonstrate how different listening contexts shape neural organization.

Both foreground and background listening contexts showed robust enhancement of within-auditory network connectivity compared to rest, suggesting that this may be a fundamental characteristic of music processing regardless of context. However, the between-network patterns diverged substantially between contexts. During concurrent cognitive task performance with background music, we observed preserved reward-motor coupling alongside reduced auditory connections to both other networks. During focused music listening, we found widespread negative correlations between motor regions and both auditory and reward networks. These patterns represent the first systematic examination of all three networks simultaneously during music engagement, revealing complex context-dependent reorganizations that were not captured by previous studies that featured pairwise networks.

### 4.1. Music Enhances Intrinsic Auditory Network Connectivity

The enhanced within-auditory network connectivity observed across both contexts aligns with increased processing demands for complex musical stimuli, extending previous findings [7,8]. This consistent finding suggests that internal auditory network coherence is a core feature of music processing. However, the between-network connectivity patterns differed markedly between contexts. During cognitive task performance with music, the auditory network showed selective decoupling from motor and reward networks, potentially reflecting efficient parallel processing that minimizes interference with concurrent task demands.

The differential connectivity patterns between MTG/ITG regions and other auditory areas during focused listening suggest hierarchical processing differences when music is the primary stimulus. These regions showed negative correlations with primary auditory areas, potentially reflecting specialized processing streams for different musical features or top-down modulation based on the listening context.

### 4.2. Context-Specific Motor and Reward Network Patterns During Music Listening

Our findings revealed predominantly negative motor-auditory and motor-reward correlations, particularly during focused music listening. This pattern appears contradictory to literature demonstrating motor activation during music listening [18,44]. However, this apparent contradiction likely reflects the specific experimental context where participants remained still during scanning. The observed patterns may represent active motor suppression rather than the absence of motor engagement with music [45,46].

During cognitive task performance with background music, motor suppression was more selective, primarily affecting the SMA and precentral gyri while preserving some motor-reward coupling. This preserved coupling might support implicit rhythm processing while maintaining task performance. These context-specific patterns highlight how experimental constraints and concurrent task demands fundamentally shape motor network involvement during music processing.

The presence of motor suppression during music listening may have broader therapeutic implications. Reduced motor excitability has been linked to high trait anxiety and depression [47,48]. Impairments in mu-wave suppression over the sensorimotor cortex have also been observed in clinical populations with atypical social adjustment, such as schizophrenia and bipolar disorder [49,50]. Moreover, many neurodegenerative disorders have motor symptoms, including rigidity, impaired balance, gait disturbances, tremors, and declining functional mobility [51,52]. These involuntary motor movements are associated with an inability to suppress motor cortex activity [53,54,55,56]. Thus, understanding when music suppresses versus enhances motor activity is essential for tailoring music-based interventions to the specific needs of the target clinical population.

### 4.3. Network Analyses Support Reward System Integration

Graph theory analyses revealed that reward regions demonstrated hub-like properties during music engagement, particularly during concurrent task performance. The increased degree, global efficiency, and betweenness centrality of regions like the NAcc, caudate, and pallidum together suggest that these areas serve as integration points during music processing. This aligns with previous findings linking NAcc connectivity to musical pleasure [26,27]. During focused music listening, the selective enhancement of amygdala and frontal orbital cortex connectivity indicates that different reward system components may be engaged depending on the listening context. The bilateral amygdala has positive correlations with auditory regions during focused listening, likely supporting its role in processing and/or evaluating the emotional content of music [23].

The distinct connectivity patterns between contexts likely reflect a combination of attentional allocation, cognitive load, motor suppression requirements, and task-specific processing demands. During cognitive task performance with music, the brain must balance multiple processing streams, potentially explaining the preserved reward-motor coupling that might facilitate dual-task coordination. The network segregation observed during focused listening could optimize music-specific processing when cognitive resources are not divided. These findings extend beyond simple attention effects, revealing how the broader cognitive and behavioral context shapes music processing networks. The differences between contexts cannot be attributed to attention alone but rather represent the complex interplay of multiple cognitive and sensory processes operating simultaneously.

### 4.4. Rethinking the Role of Auditory Connectivity in Neurorehabilitation

Our findings have implications for understanding how music functions in various real-world contexts. The preserved motor-reward coupling during cognitive task performance suggests that background music might support certain types of activities through implicit motivational and rhythmic processes. However, the reduced cross-network connectivity in this context also suggests potential limitations in music’s emotional or aesthetic impact when attention is divided.

For therapeutic applications, these context-dependent patterns suggest that intervention design should consider not just musical content but also the cognitive and behavioral context of delivery. Music presented during movement therapy might engage different neural mechanisms than music used for focused listening in emotional regulation interventions. Future reports of music-based intervention research should include the setting, delivery method, and intervention strategy [57]. For example, music presented during movement therapy [58] might engage different neural mechanisms than music used for focused listening in attention interventions [34].

### 4.5. Limitations and Future Directions

As a secondary analysis of existing datasets, we have only provided indirect evidence for distinct FC patterns across music listening contexts with varying levels of attentional engagement. The current manuscript alone cannot experimentally isolate specific factors driving the observed differences between contexts, as the contexts differed not only in attentional focus but also in concurrent cognitive demands, task requirements, and potentially participant engagement. However, similar network patterns between the Music Listening Task > Face-name Task (no-music task control) and Music Listening Task > Rest (no-music no-task control) contrasts suggest that these observed FC differences are robust to task-related demands and cognitive load, reflecting processes related to music listening rather than general task engagement.

Future research should systematically manipulate individual factors—attention, cognitive load, motor requirements, and musical features—within subjects to isolate their specific contributions to network connectivity. Studies could parametrically vary cognitive load while keeping music constant or manipulate musical features while maintaining consistent task demands. Moreover, certain musical characteristics, especially those associated with “high groove” like syncopation and strong beat salience [16,59], can evoke a pleasurable urge to move. Future studies could also manipulate these movement-inducing characteristics of music to more sensitively investigate interactions between the auditory, reward, and motor networks through varying levels of motor engagement. Understanding how individual factors such as age, clinical status, musical training, reward sensitivity, and cognitive capacity influence these context-dependent patterns would also be crucial for personalized applications.

## 5. Conclusions

This research demonstrates that music engagement involves complex reorganization of the auditory, motor, and reward networks, with patterns varying substantially across listening contexts. While enhanced within-auditory connectivity appears to be a consistent feature of music processing, between-network interactions depend on the broader cognitive and behavioral context. These findings highlight the importance of considering ecological context when studying music processing and designing music-based interventions. Rather than revealing simple attention effects, our results demonstrate that music’s neural basis is fundamentally shaped by the complex interplay of concurrent cognitive demands, motor requirements, and listening goals. This context-dependent flexibility may underlie music’s versatility as a tool for diverse applications from cognitive enhancement to emotional regulation.

## Figures and Tables

**Table 1:** Music Listening Task > Rest.

Measures	Networks	ROIs	Beta	T (Df = 29)	*p*-FDR
Degree	Reward	FOrb r	3.14	4.69	0.002931
Betweenness Centrality	Auditory Auditory	pSTG l (Cluster 1) pSTG r (Cluster 1)	−0.02 −0.03	−3.51 −3.48	0.038943 0.038943
Global Efficiency	Reward Auditory	FOrb r pSTG r (Cluster 2)	0.08 0.03	4.68 3.56	0.003016 0.032109

**Table 2:** Music Listening Task > Face-name Task.

Measures	Networks	ROIs	Beta	T (Df = 35)	*p*-FDR
Degree	Reward	Putamen l	2.06	5.01	0.001186
	Auditory	toMTGr	−2.62	−4.66	0.001186
	Motor	PostCG l	−2.63	−4.62	0.001186
	Motor	PostCG r	−2.85	−4.30	0.002133
	Reward	Putamen r	1.67	3.74	0.007853
	Reward	FOrb l	1.81	3.34	0.014927
	Auditory	pSTGr (Cluster 1)	−2.14	−3.28	0.014927
	Reward	PCC	−1.71	−3.27	0.014927
	Auditory	pITG r	1.81	3.25	0.014927
	Motor	SFG r	−1.77	−3.23	0.014927
	Reward	Caudate l	0.89	3.08	0.019980
Betweenness Centrality	Auditory	pMTG r	0.03	4.06	0.011321
	Auditory	HG r	0.03	3.84	0.011321
	Reward	Putamen r	0.03	3.78	0.011321
	Reward	IC l	0.04	3.68	0.011321
	Reward	IC r	0.04	3.61	0.011321
	Auditory	pSTG l (Cluster 1)	−0.02	−3.52	0.011801
	Motor	PostCG l	−0.01	−3.37	0.015142
	Motor	PostCG r	−0.01	−3.30	0.015694
	Auditory	pSTG r (Cluster 1)	−0.02	−3.25	0.015922
	Reward	Putamen l	0.03	2.87	0.036908
	Auditory	pMTG l	0.04	2.81	0.039231
	Reward	NAcc l	−0.01	−2.75	0.041886
Clustering Coefficient	Motor	PostCG r	0.17	5.19	0.000743
	Auditory	pSTG l (Cluster 1)	0.11	4.64	0.001540
	Auditory	pSTG r (Cluster 1)	0.10	3.79	0.010476
	Motor	PostCG l	0.12	3.59	0.013373
	Reward	toITG l	0.15	3.50	0.016786
Local Efficiency	Auditory	pSTG r (Cluster 1)	0.08	4.76	0.001662
	Motor	PostCG r	0.10	4.64	0.001662
	Auditory	toITG l	0.19	4.04	0.006698
	Auditory	pSTG l (Cluster 1)	0.07	3.85	0.006698
Global Efficiency	Reward	Putamen l	0.11	6.02	0.000074
	Reward	Putamen r	0.10	5.55	0.000134
	Reward	Pallidum l	0.09	5.31	0.000175
	Reward	FOrb l	0.05	5.03	0.00029
	Reward	FOrb r	0.05	4.76	0.000483
	Reward	Pallidum r	0.07	4.54	0.000734
	Reward	Caudate l	0.09	4.39	0.00095
	Reward	IC l	0.05	4.10	0.00185
	Reward	Caudate r	0.08	3.81	0.003603
	Auditory	pITG l	0.05	3.77	0.003603
	Motor	PostCG r	−0.06	−3.67	0.004384
	Auditory	pSTG l (Cluster 2)	0.07	3.62	0.004554
	Motor	PostCG l	−0.05	−3.55	0.00506
	Reward	NAcc l	0.08	3.38	0.007391
	Auditory	HG r	0.03	3.01	0.017139
	Auditory	aSTG r	0.03	2.99	0.017139
	Auditory	HG l	0.04	2.85	0.022859
	Auditory	toMTGr	−0.02	−2.76	0.026796
	Auditory	pSTG r (Cluster 1)	−0.02	−2.62	0.034589
	Auditory	pSTG r (Cluster 2)	0.02	2.61	0.034589
	Auditory	pMTG r	0.02	2.58	0.03509
	Reward	NAcc r	0.07	2.53	0.038252

**Table 3:** SART and WM combined > Rest.

Measures	Networks	ROIs	Beta	T (Df = 27)	*p*-FDR
Degree	Reward	IC r	−2.46	−4.84	0.002273
	Reward	Caudate l	1.12	3.78	0.012348
	Reward	NAcc l	0.57	3.7	0.012348
	Reward	NAcc r	0.41	3.69	0.012348
	Reward	IC l	−1.83	−3.45	0.018291
	Auditory	pITG r	−1.35	−3.19	0.022754
	Reward	Pallidum r	0.17	3.15	0.022754
	Motor	SMA l	−1.34	−3.14	0.022754
	Motor	midFG l	1.43	3.13	0.022754
	Auditory	pSTG r (Cluster 2)	0.98	3.03	0.025982
	Auditory	aITG r	−1.22	−2.99	0.025982
Clustering Coefficient	Auditory	pSTG r (Cluster 1)	0.13	4.48	0.004509
Local Efficiency	Auditory	pSTG r (Cluster 1)	0.10	4.17	0.010295
Global Efficiency	Motor Auditory Auditory Reward Motor Reward Motor Reward Auditory Reward Reward Motor Reward	midFG l pMTG l (Cluster 1) pSTG r (Cluster 2) NAcc l midFG r Pallidum r PreCG l Caudate l toITG r IC r FOrb l ACC NAcc r	0.03 0.02 0.09 0.05 0.02 0.04 −0.02 0.06 0.02 −0.02 0.03 0.02 0.05	4.73 4.12 3.81 3.35 3.25 3.19 −3.08 3.07 2.78 −2.72 2.71 2.7 2.68	0.003054 0.007821 0.012025 0.029272 0.029273 0.029273 0.029273 0.029273 0.046373 0.046373 0.046373 0.046373 0.046373

## Data Availability

The raw data supporting the conclusions of this article will be made available by the authors on request.

## Supplementary Materials

The following supporting information can be downloaded at: https://www.mdpi.com/article/10.3390/brainsci16010015/s1, Supplementary S1: List of Researcher-Selected Song Stimuli, Supplementary S2: Within-Task ROI-to-ROI Connectivity Matrices.
