# Supplementary material for: Functional Connectivity of Auditory, Motor, and Reward Networks at Rest and During Music Listening
Source: Brain Sci. 2025 Dec 22;16(1):15. doi: 10.3390/brainsci16010015 (PMC12839246; doi:10.3390/brainsci16010015)
Supplement: Supplementary file 1 [file brainsci-16-00015-s001.zip › S1.pdf]

Supplementary S1: List of Researcher-Selected Song Stimuli

Table S1

Foreground Listening Group

Song Title	Artist/Composer	Music Selection
Adagio for Strings	Barber	Well-Known
Symphony No. 2, Finale	Mahler	Well-Known
Cavatina Op. 130	Beethoven	Well-Known
John Wayne Gacy Jr.	Sufjan Stevens	Well-Known
Hurt	Johnny Cash	Well-Known
Sound of Silence	Simon and Garfunkel	Well-Known
Reckoner	Radiohead	Well-Known
Nocturne in C Sharp Minor	Chopin	Well-Known
Nessun Dorma	Andrea Bocelli	Well-Known
Symphony No. 6, Movement 1	Tchaikovsky	Well-Known
Bohlen-Pierce Pan Flute Folk Tune*	Arturo Grolimund	Novel/BP
Hoquetus II*	Johannes Kretz	Novel/BP
Reminiscences*	Steven Yi	Novel/BP
Beyond The Horizon*	Georg Hajdu	Novel/BP
When the Moon Casts Shadows*	Hubert Ho	Novel/BP
Simmer*	Hubert Ho	Novel/BP
Vertigo Temporum*	Hubert Ho	Novel/BP
Manual Labor*	Hubert Ho	Novel/BP

Note: This song list is the same list used in Quinci et al. (2022) and Belden et al. (2023). Out of the 24 total song stimuli used in the music-listening fMRI task, there are 10 well-known songs and 8 novel Bohlen-Pierce (BP) songs. *Only n=12 participants heard these 8 BP song clips; the remaining n=27 participants heard 8 clips of BP melodies played on the clarinet (original stimuli from Kathios & Sachs et al., 2023).

Table S2

Background Listening Group

Song Title	Artist(s)
(I've Had) The Time of My Life	Bill Medley and Jennifer Warnes
Africa	Toto
All Of Me	John Legend
Beautiful	Christina Aguilera
Because Of You	Kelly Clarkson
Complicated	Avril Lavigne
Eye of the Tiger	Survivor
Girl on Fire	Alicia Keys
Hallelujah	Rufus Wainwright
Hello	Adele

Hey There Delilah	The Pain White Ts
I Have Nothing	Whitney Houston
I Will Always Love You	Whitney Houston
I Will Follow You Into The Dark	Death Cab For Cutie
I'll Make Love To You	Boys 2 Men
Issues	Julia Michaels
Just Give Me A Reason	P!NK
Need You Now	Lady Antebellum
Not Gonna Write You A Love Song	Sara Bareilles
Perfect	Ed Sheeran
Praying	Kesha
See You Again	Charlie Pluth
Shallow	Lady Gaga
Since You Been Gone	Kelly Clarkson
Someone Like You	Adele
Stay With Me	Sam Smith
Thinking Aloud	Ed Sheeran
Versace On The Floor	Bruno Mars
When I Was Your Man	Bruno Mars
Wrecking Ball	Miley Cyrus

Note: Each participant received a randomized song list. They listened to 6 songs per task (half modulated and the other half unmodulated), 12 songs in total. Participants provided liking and familiarity ratings for all songs.
