# Supplementary material for: Functional Connectivity of Auditory, Motor, and Reward Networks at Rest and During Music Listening
Source: Brain Sci. 2025 Dec 22;16(1):15. doi: 10.3390/brainsci16010015 (PMC12839246; doi:10.3390/brainsci16010015)

Supplementary S2: Within-Task ROI-to-ROI Connectivity Matrices

Study 1: Foreground Music Group *– Resting State*

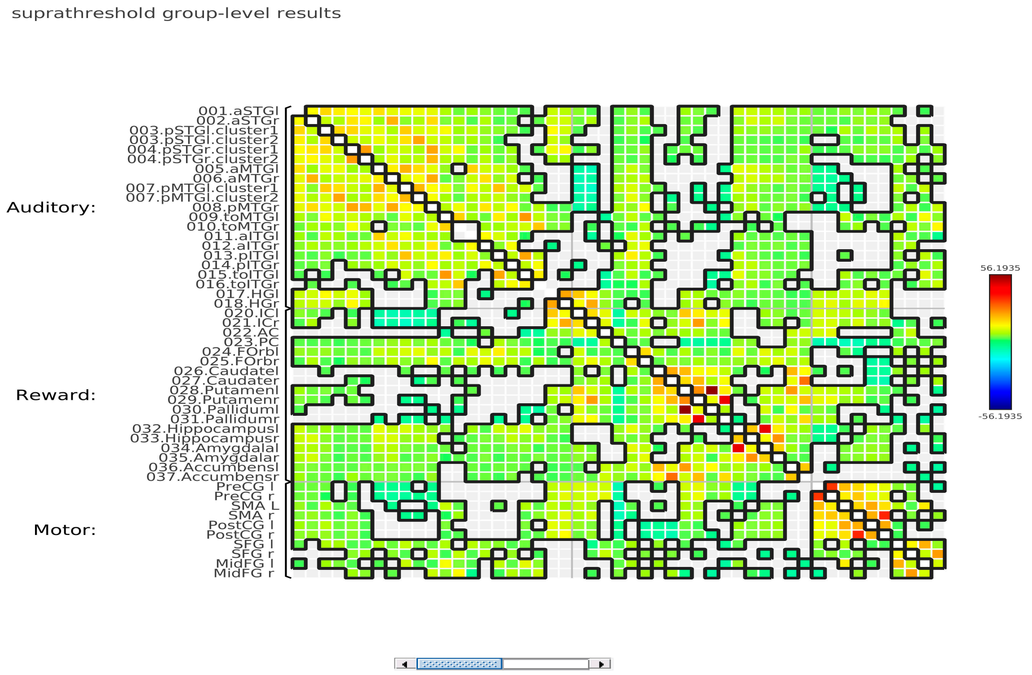

Study 1: Foreground Music Group *– Music Listening Task*

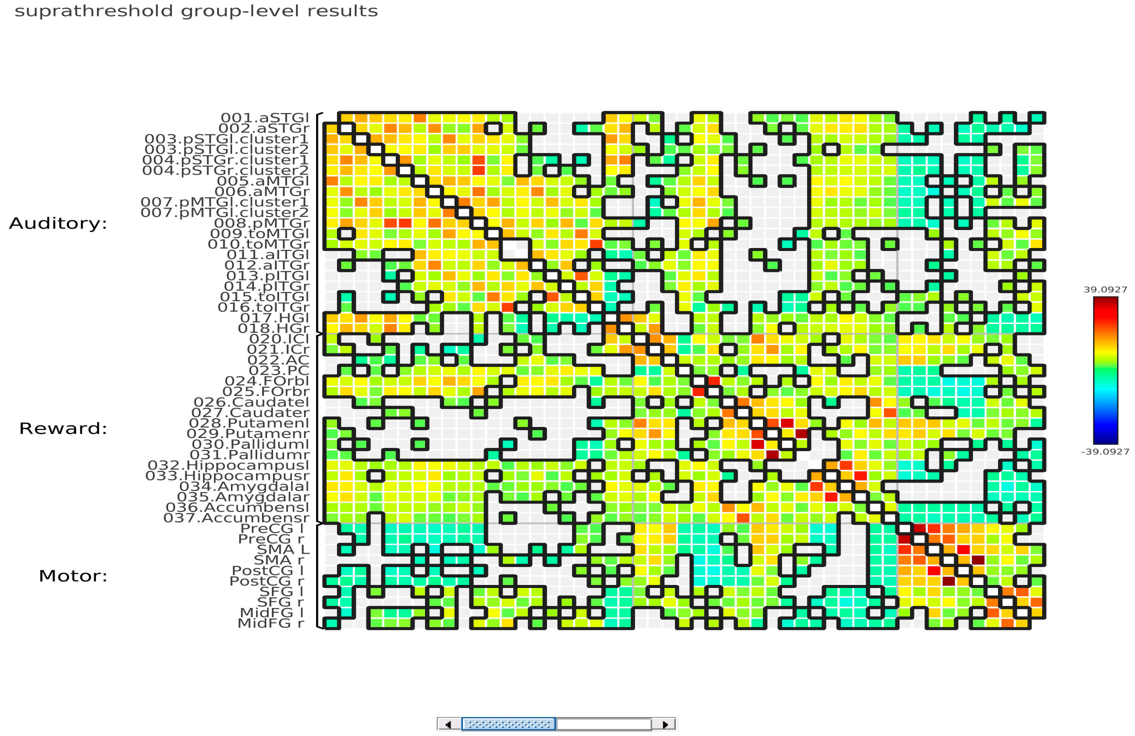

Study 1: Foreground Music Group *– Face-name Task*

suprathreshold group-level results

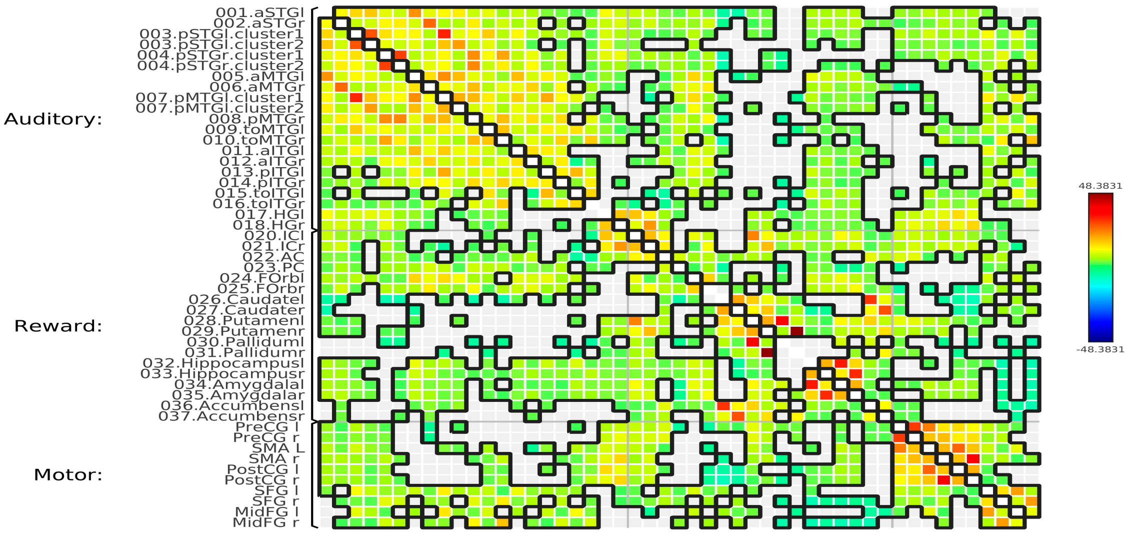

***Study 2: Background Music Group** – Resting State*

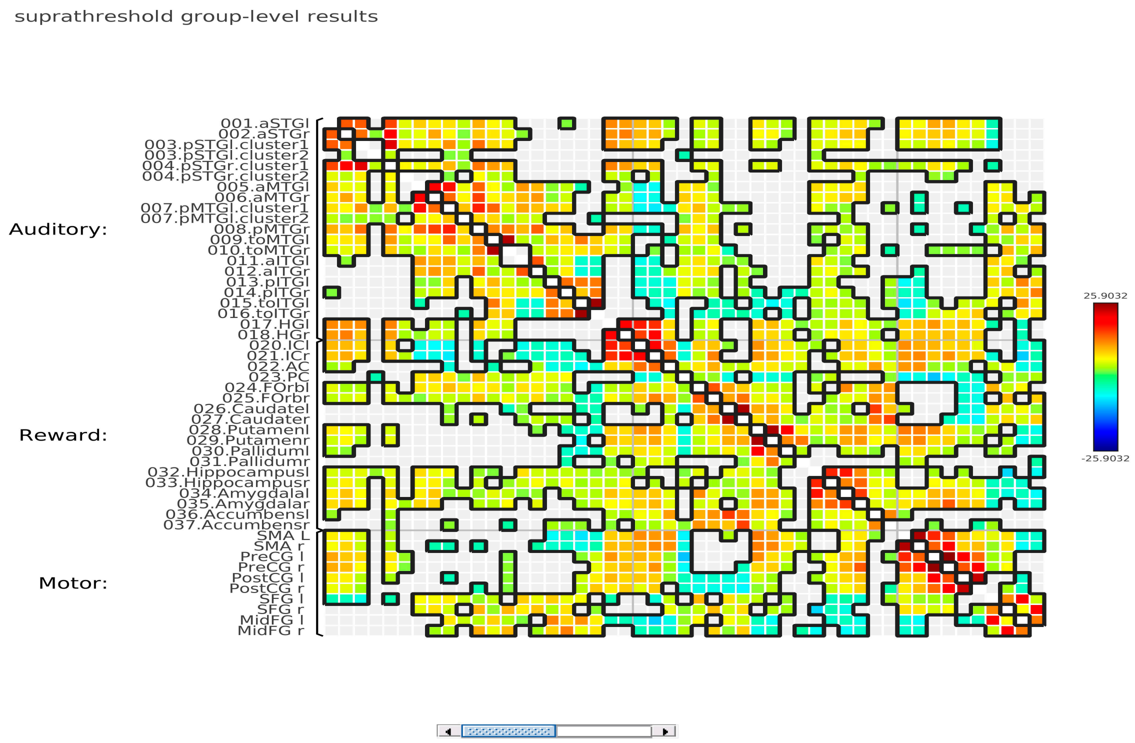

***Study 2: Background Music Group** – SART and WM Combined*

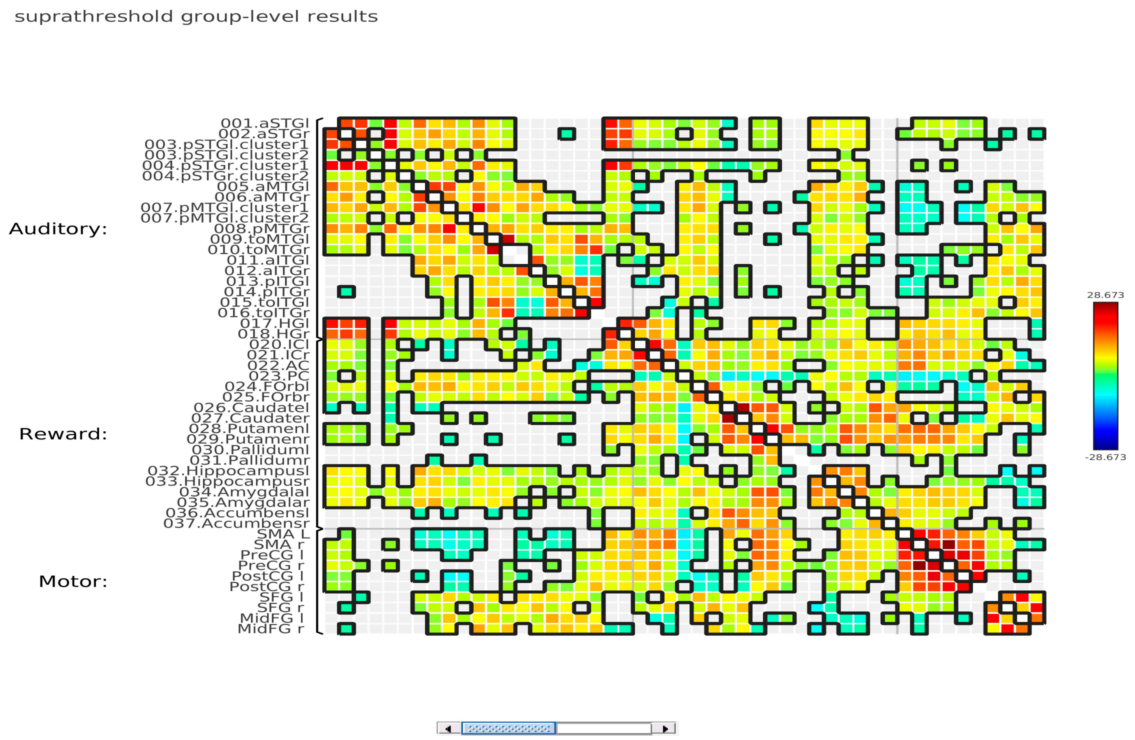

Supplement: Supplementary file 1 [file brainsci-16-00015-s001.zip › S2.pdf]
